# Vein Graft Preservation During Coronary Artery Bypass Graft Surgery: Operative Techniques, Biomaterials and Advances from Tissue Engineering

**DOI:** 10.3390/jfb17060305

**Published:** 2026-06-20

**Authors:** Benedetto Ferraresi, Antonio Nenna, Mohamad Jawabra, Diletta Corrado, Filippo Barberi, Carmelo Dominici, Giovanni Casali, Lucia Barbieri, Gabriele Tumminello, Stefano Carugo, Massimo Chello, Mario Lusini

**Affiliations:** 1 Cardiac Surgery, Fondazione Policlinico Universitario Campus Bio-Medico, Via Alvaro del Portillo 200, 00128 Rome, Italy; benedetto.ferraresi@unicampus.it (B.F.); mohamad.jawabra@unicampus.it (M.J.); diletta.corrado@unicampus.it (D.C.); f.barberi@policlinicocampus.it (F.B.); m.chello@policlinicocampus.it (M.C.); m.lusini@policlinicocampus.it (M.L.); 2 Department of Cardio-Thoracic-Vascular Diseases, Foundation IRCCS Ca’ Granda Ospedale Maggiore Policlinico, Via Francesco Sforza 28, 20122 Milan, Italy; carmelo.dominici@policlinico.mi.it (C.D.); lucia.barbieri@policlinico.mi.it (L.B.); gabriele.tumminello@policlinico.mi.it (G.T.); stefano.carugo@policlinico.mi.it (S.C.); 3 Cardiac Surgery, Azienda Ospedaliero Universitaria Maggiore della Carità di Novara, Corso Mazzini 18, 28100 Novara, Italy; giovanni.casali@maggioreosp.novara.it; 4 Department of Clinical Sciences and Community Health, Università degli Studi di Milano, Via Festa del Perdono 7, 20122 Milan, Italy

**Keywords:** vein graft, failure, scaffold, support, coronary artery bypass graft

## Abstract

The failure of saphenous vein grafts following coronary artery bypass grafting (CABG) remains a significant issue, as it limits the durability of vein-based surgical revascularisation compared to arterial conduits. Venous graft disease is a dynamic process that begins early in the perioperative period as a consequence of harvesting trauma, ex vivo preservation, and the sudden exposure of the conduit to the arterial haemodynamic environment. This narrative review summarises the available evidence on local graft protection strategies, focusing on intraoperative and perioperative approaches aimed at preserving endothelial integrity, attenuating initial inflammation, and limiting maladaptive remodelling. Specifically, the review analyses the role of endothelium-protective preservation solutions, external support devices, biodegradable drug-eluting biomaterials, and locally targeted RNA therapies. Preclinical and early clinical evidence suggests that local graft protection is biologically plausible and may reduce intimal hyperplasia, luminal irregularity and adverse graft remodelling. However, its impact on long-term clinical outcomes remains uncertain. An integrated approach combining harvest optimisation, conduit preservation, mechanical support, and local delivery of drugs or regulatory molecules may represent a promising framework for improving vein graft biology; however, its ability to translate into durable patency gains and improved clinical outcomes after CABG requires further clinical validation.

## 1. Introduction

Coronary artery bypass grafting (CABG) remains a cornerstone therapy for complex coronary artery disease, and the saphenous vein graft (SVG) is used in the vast majority of procedures to revascularise ‘non-LAD targets’ (i.e., not the Left Anterior Descending artery). However, the durability of SVGs continues to lag behind that of arterial conduits. Long-term attrition of SVGs remains common and has clinically meaningful consequences, including recurrent angina, myocardial infarction and the need for repeat revascularisation [1,2]. Vein graft disease (VGD) is recognised as a time-dependent biological response to surgical handling and to abrupt exposure of a venous conduit to the arterial environment [3]. As systemic secondary prevention alone cannot fully neutralise a locally initiated process, the field has progressively moved towards local graft protection, with interventions applied directly to the conduit before or at implantation, to preserve endothelial integrity, blunt early inflammation and modulate remodelling [4]. The operating room provides a unique opportunity for translation: the autologous graft is temporarily outside the body and can be treated ex vivo, enabling high local exposure with minimal systemic toxicity [5]. Intraoperative storage conditions can measurably influence endothelial structure and function, and non-physiological media (classically, simple saline) can be detrimental, motivating the adoption of buffered, antioxidant or otherwise protective preservation strategies [6]. In parallel, dedicated in vivo preservation solutions have been developed with reduced oxidative stress/inflammation and attenuation of intimal hyperplasia, supporting the broader premise that the way in which we handle and store the vein has biological significance [7]. Contemporary pathophysiology frameworks emphasize that SVG failure is often initiated intraoperatively through endothelial denudation, excessive mechanical stretch and intimal/adventitial injury, and those insults amplify thrombogenicity and inflammatory signalling during reperfusion. Consequently, standardisation of harvesting and handling has become a central theme in prevention strategies [8]. Another key area of focus is external support and perivascular delivery platforms, which are designed to reduce adverse wall stress while enabling local pharmacology. External stents and wraps have progressed to the stage of clinical evaluation; for example, randomised trials have examined the effects of external stenting on graft remodelling and disease progression. Beyond permanent supports, biodegradable approaches aim to provide temporary scaffolding during the high-risk remodelling phase [9]. An even more ‘active’ strategy involves converting the perivascular space into a drug depot. A time-programmed, multiple-drug-eluting external sheath has been reported to suppress neointimal hyperplasia in experimental vein grafting. This illustrates how device engineering may translate mechanistic targets into controllable local dosing [10].

## 2. Methods

This article was designed as a narrative review. A non-systematic literature search was conducted in PubMed/MEDLINE, Scopus, Google Scholar, and the Cochrane Library to identify relevant studies on saphenous vein graft preservation during coronary artery bypass grafting. The search focused on combinations of terms including saphenous vein graft, coronary artery bypass grafting, vein graft failure, preservation solution, DuraGraft, no-touch harvesting, external stent, external support, biomaterials, drug-eluting sheath, RNA therapy, microRNA, endothelial injury, intimal hyperplasia, and tissue engineering. The review considered experimental, translational, and clinical studies, as well as selected review articles, when they were deemed relevant to the aims of the manuscript. Priority was given to publications addressing intraoperative and perioperative local graft-protection strategies, including atraumatic harvesting, conduit preservation, external mechanical support, biomaterial-based platforms, and locally delivered molecular therapies. Reference lists of key articles were also screened manually to identify additional pertinent studies. Studies were selected on the basis of their relevance to the biological mechanisms and clinical implications of vein graft disease prevention rather than through a formal systematic-review process. Articles not directly related to saphenous vein graft preservation in the setting of surgical revascularisation, duplicate reports, and publications with limited relevance to the predefined thematic scope were not considered further. The final evidence was synthesized qualitatively and organized into thematic sections covering operative techniques, preservation media, external support technologies, biomaterial-enabled local drug delivery, and emerging tissue-engineering and RNA-based approaches. Given the narrative nature of the review, no formal risk-of-bias assessment or meta-analysis was performed.

## 3. Vein Graft Disease in Surgical Revascularisation: Clinical Burden and Impact

### 3.1. Epidemiology and Clinical Consequences of Saphenous Vein Graft Failure

Despite growing interest in multiple arterial grafting, the SVG remains the primary conduit for non-LAD (left anterior descending artery) targets, accounting for nearly 95% of contemporary CABG procedures. This widespread utilisation makes SVG failure a major determinant of long-term outcomes after CABG. Importantly, SVG attrition translates into a substantial downstream need for repeat revascularisation: approximately 13% of CABG patients undergo repeat revascularisation within 10 years, and patients with a history of CABG account for ~18% of all percutaneous coronary interventions (PCIs), with ~6% of all PCIs being performed on SVGs, highlighting how frequently late graft disease drives recurrent ischemia and re-intervention [2]. From a procedural standpoint, treating degenerated SVGs is often challenging. SVG PCI is limited by complications such as no-reflow, as well as a high incidence of restenosis during follow-up. Consequently, PCI of the corresponding native coronary artery is often favoured over SVG stenting when feasible, while redo-CABG is typically associated with a higher perioperative risk than PCI and is generally reserved to treat LAD disease resulting from atherosclerotic progression or technical failure at index procedure [2]. Together, these considerations emphasize that preventing SVG failure is not just an anatomical goal, but also a strategy with direct implications for symptoms, events and healthcare utilisation.

### 3.2. Disease Timeline (Early Thrombosis, Intimal Hyperplasia, Late Atherosclerosis)

VGD is classically conceptualized as a time-dependent continuum beginning with perioperative injury and evolving through distinct biological phases.

The early phase (within 30 days) involves thrombosis and technical and rheological failure. Early graft failure, which occurs within the first month, is primarily attributed to technical factors relating to harvesting and handling, as well as rheological issues; thrombosis is the dominant mechanism in this phase. This phase is particularly susceptible to endothelial disruption and ischaemia–reperfusion injury incurred during conduit procurement and storage.

The intermediate phase (1 month to 1 year) is characterized by intimal hyperplasia (“arterialization”). As the vein adapts to the arterial environment, the graft undergoes chronic inflammatory remodelling. Intimal hyperplasia is a process that starts with endothelial cell activation, followed by smooth muscle cell migration and proliferation, and extracellular matrix remodelling. This creates the substrate for later luminal compromise [2].

The late phase (>1 year) is characterised by accelerated atherosclerosis and superimposed thrombosis. Beyond the first year, SVGs often develop superimposed atherosclerosis on the background of intimal hyperplasia, leading to progressive stenosis and late graft occlusion. In this context, ‘late failure’ (occurring after 30 days and extending for years) is considered the clinical manifestation of VGD driven by ongoing inflammation, remodelling and atherothrombotic progression.

**Figure 1:**
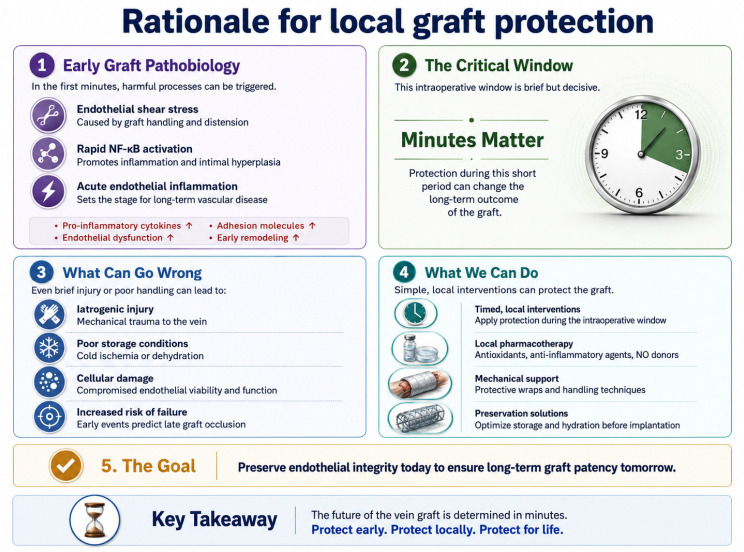
Rationale for local graft protection. The timing labels shown in the figure are schematic conceptual markers intended to illustrate the general sequence of early perioperative events in vein graft disease, rather than precise timepoints directly derived from any single cited study.

The rationale for local graft protection is that the decisive pathobiology of graft failure begins immediately, often while the conduit is still ex vivo, making it easily modifiable (Figure 1). Arterialization rapidly exposes the endothelium of the saphenous vein to arterial shear stress, prompting the release of pro-inflammatory signals within minutes. NF-κB activation can be detected after around 30 min. Pharmacological inhibition of NF-κB attenuates these acute responses, suggesting that early, localized interventions could prevent the onset of graft inflammation. A second critical component is iatrogenic and potentially preventable: intraoperative manipulation and preservation conditions can compromise graft viability, which can deteriorate after as little as 15 min in saline. In contrast, it can be maintained for hours using specialized solutions (e.g., DuraGraft^®^). In animal models, for instance, a biodegradable external wrap improves lumen uniformity and shear stress, thereby reducing overdistension, wall thickening, intimal hyperplasia and inflammatory signatures. In summary, because VGD follows a predictable timeline and the earliest signs are concentrated in the perioperative window, the operating theatre provides a unique opportunity for interventions that target the graft and can potentially influence long-term outcomes [8] (Table 1).

## 4. Intraoperative and Perioperative Graft Protection Strategies

### 4.1. No-Touch and Soft-Touch Vein Harvesting

Among surgical strategies aimed at reducing early conduit injury, no-touch and soft-touch saphenous vein harvesting deserve specific consideration. The no-touch technique preserves the vein and its surrounding perivascular tissue to limit endothelial damage, reduce overdistension and maintain more physiological mechanical support for the graft. Available randomised evidence suggests that this approach may improve early and mid-term graft patency compared with conventional open harvesting. However, the magnitude of this benefit appears relatively modest and has not consistently translated into a clear reduction in major adverse clinical events in the currently available studies. Conversely, open no-touch harvesting has been associated with a higher incidence of leg wound complications. These observations suggest that atraumatic conduit procurement is biologically important and that the balance between graft protection and surgical-site morbidity must be carefully considered. In this context, soft-touch harvesting and minimisation of mechanical trauma and excessive distension may be pragmatic strategies for preserving graft integrity while limiting the disadvantages associated with more extensive tissue harvesting [20].

### 4.2. Preservation Solutions: Buffered Versus Saline and “Endothelial-Friendly” Approaches

During CABG, the harvested saphenous vein inevitably experiences an ischaemic period between its removal and implantation. Therefore, the choice of storage solution is not a trivial logistical detail, but rather a modifiable factor that can influence early endothelial injury and subsequent vein graft disease. Current practice shows substantial heterogeneity: a survey of storage practices across high-volume centres found that over half still use either heparinised blood or normal saline for intraoperative storage, while a smaller proportion use buffered solutions specifically designed to preserve endothelial function [9]. Blood is often used as a preservation medium. Unlike circulating blood, which is subjected to arterial and venous pressure, extracorporeal blood is subjected to atmospheric pressure. This results in a loss of partial CO_2_ pressure, causing a rapid increase in blood pH and making it alkaline. Studies have shown that smooth muscle and endothelial cells lose vitality even after short-term exposure to slightly alkaline solutions [14]. Despite its widespread adoption, normal saline is increasingly recognised as being biologically suboptimal for vascular conduits. The rationale is mechanistic: non-buffered, non-ion-balanced solutions can promote endothelial dysfunction, oxidative stress, and the loss of nitric oxide bioavailability—processes that can occur even with relatively short exposure times [9]. These considerations have driven the development of ‘endothelial-friendly’ preservation solutions, which are typically pH-buffered, ionically balanced and supplemented with antioxidants and nitric oxide-supporting components. One example is DuraGraft^®^, a pH-buffered, ionically balanced physiological solution containing salts and antioxidants such as L-glutathione, L-ascorbic acid and L-arginine (a nitric oxide synthase substrate), as well as other additives [9]. The key concept beyond formulation is single-use, intraoperative treatment aimed at mitigating ischemia/reperfusion injury (IRI) of the conduit endothelium. In other words, it involves intervening during the perioperative ‘window’ when injury is initiated, but before irreversible, downstream remodelling is established [9]. Interestingly, the potential relevance of conduit preservation may extend beyond the graft wall itself. A retrospective propensity-matched study of patients undergoing on-pump coronary artery bypass grafting (CABG) found that repeated graft flushing with DuraGraft during distal anastomotic leak testing was associated with lower postoperative high-sensitivity troponin I release compared with saline/Biseko. This suggests a possible adjunctive myocardial protective effect mediated by the downstream intracoronary delivery of the preservation solution. However, these findings should be interpreted with caution, given that the study was single-center, retrospective and not powered to detect differences in clinical outcomes [21].

### 4.3. External Support of Vein Grafts as a Local Graft Protection Strategy

**Figure 2:**
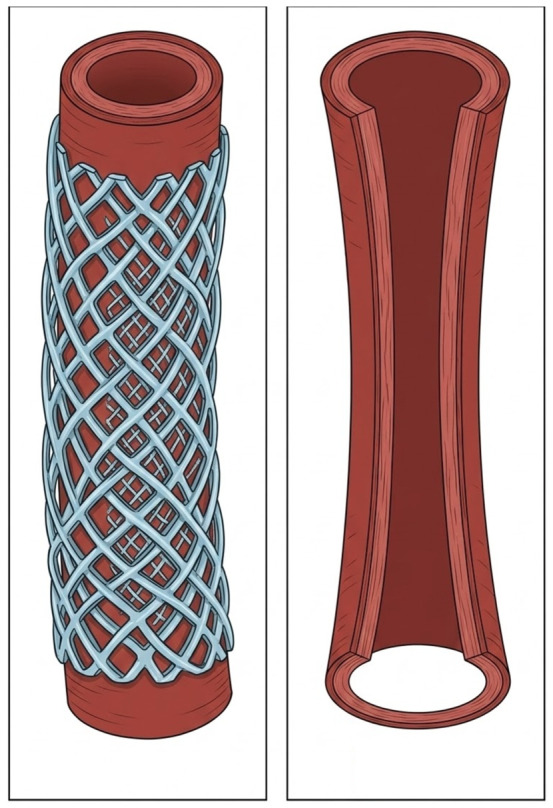
Schematic representation of the VEST external support platform applied to a saphenous vein graft (**left**), shown in comparison with a conventional unsupported graft (**right**). The figure is intended as an illustrative representation of the VEST device rather than a generic external support concept.

In the coronary setting, the most extensively studied clinical external support device is the VEST, an external metal mesh designed to stabilize the saphenous vein following implantation by reducing wall tension and flow disturbances (Figure 2). The VEST III randomised, multicenter study found that, after two years, there was no significant difference in overall patency between stented and non-stented saphenous vein grafts (SVGs) (78.3% vs. 82.2%). However, the supported grafts showed a better angiographic profile and a significant reduction in intimal hyperplasia, indicating a benefit on surrogate remodelling endpoints rather than definitive patency improvement [11]. The patency rate of stented SVGs was 98% at 6–12 months, and more importantly, there was a marked difference in lumen uniformity on CTA: 90% of stented grafts were uniform, compared to 37% of non-stented grafts. This supports the hypothesis that the primary benefit lies in geometric remodelling rather than early patency [11].

### 4.4. Devices and Biomaterials for External Protection and Local Drug Delivery

From a large-animal preclinical perspective, a braided device consisting of four 1 cm strips of Vicryl-Rapide surgical mesh woven in a ‘finger-trap’ architecture to allow adjustment for a snug, custom fit around the graft has been proposed to reduce the haemodynamic impact on the graft [9]. This design uses a mesh material that is reported to degrade in vivo after approximately two weeks, providing temporary external support during the initial remodelling period. After four months, the authors reported that the device had completely degraded in the wrapped grafts. They also noted that all the grafts in the Wrap group remained patent, whereas the No-Wrap group included one with a severe aneurysm and one with severe stenosis [9]. Using MRI-informed computational fluid dynamics (CFD), Ramachandra et al. demonstrate that the wall shear stress distributions in the ‘No-Wrap’ group were frequently either too low or too high, whereas the ‘Wrap’ group exhibited distributions that were more consistent with improved haemodynamic normalization [9]. The Wrap group displayed a more uniform distribution of luminal areas compared to the broad variability observed without external support and histologically, wrapped grafts demonstrated reduced neointimal hyperplasia and improved luminal uniformity compared with untreated grafts [9].

Bulk RNA sequencing and pathway analysis revealed that wrapped grafts exhibited reduced enrichment of inflammatory signalling networks, including pathways such as HIF-1, MAPK, NF-κB, TNF and IL-17. Conversely, genes associated with endothelial function, such as PECAM1, NOS3 and KDR, were found to be upregulated in wrapped grafts compared to controls. Although external wrapping has shown promise in preclinical models, the authors discuss that clinical results with external stenting/wrapping have not consistently demonstrated superior long-term patency [15]. They suggest that the use of stiff, non-conforming, non-degradable materials might contribute to inconsistent performance and support the investigation of biodegradable approaches [9,22,23].

### 4.5. Time-Programmed External Sheaths for Sequential Drug Delivery

In a preclinical biomaterials study, Zhang et al. described the development of a “time-dependent, slow-release, multiple-drug-eluting external sheath” designed to align with the phase-dependent evolution of pathology in saphenous SVG failure [10]. The sheath incorporates three agents with distinct release timelines (Fasudil dihydrochloride, 100% released after 63 days; everolimus, 100% released after 84 days; simvastatin one third released at 20 weeks, indicating prolonged delivery). The authors explicitly frame this design as an attempt to provide long-term inhibition through staged release behaviour. The sheath is fabricated using multi-channel and coaxial electrospinning to combine polymers with distinct degradation behaviours, generating differential release kinetics [10]. In a four-month rabbit jugular vein graft model, Zhang et al. found that the grafts remained patent across the groups, whereas the controls showed progressive dilation and altered flow ratios. The drug-eluting sheath reduced intimal hyperplasia and produced lower intima/media (I/M) ratios than the control and non-drug ‘C-sheath’ groups [10]. They reported that, while mechanical constraint alone may delay NIH progression, it does not block it, and that the drug-loaded sheath reduced proliferative and smooth muscle markers, being associated with improved endothelial recovery readouts, including increased eNOS [24].

### 4.6. Intraoperative Ex-Vivo Graft Conditioning

The PREVENT IV programme provides the main randomised clinical example of an alternative to external support strategies, focusing on intraoperative graft handling and ex vivo conditioning prior to implantation. In phase III of the PREVENT IV trial, autologous saphenous vein grafts were treated ex vivo with edifoligide—an E2F transcription factor decoy designed to inhibit neointimal hyperplasia—using a pressure-mediated delivery system which exposed the conduit to low, non-distending pressure (6 psi) for ten minutes prior to implantation. The aim of this drug–device combination was to achieve local transfection of the vein graft wall during the intraoperative window. This strategy was intended to target early biological mechanisms of graft failure, rather than to provide prolonged post-implant mechanical support or sustained external drug release [25]. Previous studies had indicated the potential biological and structural advantages of this approach, and the PREVENT IV trial was specifically designed to determine whether ex vivo vein graft pretreatment could enhance angiographic patency and long-term clinical outcomes following CABG. However, subsequent analyses from the PREVENT IV population showed that edifoligide itself did not reduce SVG failure or secondary clinical endpoints compared with placebo. In contrast, a clinically relevant signal emerged regarding graft preservation practices: vein grafts stored intraoperatively in buffered saline solution had significantly lower failure rates at one year than grafts preserved in saline solution or blood, although this observation derived from secondary/post hoc clinical analyses rather than the primary randomised comparison. There was also a trend towards improved five-year outcomes, suggesting that handling of the conduit during the perioperative period may be at least as important as targeted antiproliferative delivery in determining subsequent graft durability [19]. A post hoc analysis of the PREVENT-IV trial investigating the use of the radial artery provides additional insight into these findings by showing that the choice of conduit remains a major determinant of outcome. In that cohort, one-year failure rates were comparable for radial artery grafts and saphenous vein grafts, but higher than for LIMA grafts. However, five-year clinical outcomes were similar. Notably, radial graft failure occurred more frequently when bypassing moderately stenotic targets, which is consistent with competitive flow. Taken together, these studies suggest that improving CABG conduit performance will likely require an integrated strategy combining careful conduit selection with atraumatic harvesting and optimised preservation solutions. Where effective, this should be combined with local biological modulation of the graft wall [12].

### 4.7. RNA Therapies: From Pathway Targeting to Feasible Local Delivery

The included literature on RNA-based strategies is still largely mechanistic and preclinical, but it illustrates how target identification can be connected to delivery feasibility in the CABG setting. Spatial transcriptomic profiling identified the early activation of mechanistically relevant and potentially ‘druggable’ pathways, including NF-κB signalling and cytokine/inflammatory cascades [26]. NF-κB, in particular, has been identified as a viable therapeutic target by Ward et al., who demonstrated that NF-κB inhibition prevents acute shear stress-induced endothelial activation in human saphenous veins in vitro, thereby reducing inflammatory activation and monocyte adhesion [27]. This work is important because it links arterial shear stress—an unavoidable stimulus at implantation to a defined, modifiable signalling axis [28,29]. Crucially, Ward et al. presented CABG as a setting in which localised endothelial-directed therapies could be employed. They discussed approaches such as cis-element decoy oligonucleotides and siRNA-mediated knockdown, as well as gene transfer strategies, to suppress NF-κB signalling [13]. The surgical ‘window of access’ concept is further supported by Qu et al.’s miRNA study, which showed that upregulation of miR-126-3p promotes saphenous vein endothelial cell proliferation and inhibits intimal hyperplasia. This is mechanistically linked to SPRED-1 and PIK3R2 signalling. From a translational perspective, the most relevant finding is that Qu et al. demonstrated that pretreating the local graft prior to implantation confines the therapeutic signal to the graft (Cy3 labelling), thereby improving re-endothelialisation and reducing intimal thickening [27]. Together, these RNA-focused studies support a coherent pathway involving the identification of early injury under arterial conditions, the selection of targets linked to endothelial activation and remodelling, and the local delivery of these targets during the perioperative window in order to avoid systemic exposure and maximise the effects of the graft [26,27]. Overall, the available evidence supports a layered approach rather than reliance on a single intervention, but rather a layered local protection strategy that matches the timing and mechanism. This strategy should minimise iatrogenic injury through standardised harvesting and avoidance of preventable mechanical insults (e.g., clipping-related stenosis patterns), while optimising IRI using endothelial-protective storage strategies. These strategies are supported by improvements in function and biomarkers in experimental models [16]. The use of RNA-based approaches to target early mechano-transductive signalling appears promising, based on human early-response transcriptomics and validated by ex vivo functional inhibition studies and demonstrations of graft-confined delivery [26,27].

## 5. Clinical Implications

A consistent theme across the included studies is that the earliest events of VGD are initiated during the perioperative period, specifically during harvesting, storage, and the abrupt transition to arterial haemodynamics. However, evidence in support of this concept comes from mechanistic studies in humans and preclinical models, as well as a more limited body of clinical data. Spatial transcriptomic profiling of human saphenous veins exposed to arterial conditions ex vivo has revealed that venous tissue exhibits a pronounced pro-inflammatory and remodelling signature within four hours. This includes activation of the NF-κB, TNF, MAPK and PI3K/Akt pathways, leukocyte adhesion signalling, and early matrix and wound-healing programmes [26]. These responses are also spatially and cell-type specific. For example, endothelial clusters upregulate inflammatory mediators such as IL6, IL1B, CXCL8 and SELE. Meanwhile, smooth muscle cells and fibroblasts exhibit activation patterns consistent with migration, proliferation, and extracellular matrix remodelling. All of these features are central to intimal hyperplasia and maladaptive graft adaptation [26]. Together, these human data provide strong mechanistic support for the concept that local intervention during the initial transition to arterial conditions can alter subsequent graft disease progression by targeting the stage at which inflammatory programmes are first established. Among the perioperative variables that may influence this process, conduit preservation and handling are important modifiable determinants of outcome. However, surveys of intraoperative storage practices continue to reveal significant heterogeneity, with saline and heparinised blood still being widely used despite concerns about endothelial injury and the absence of physiological buffering [8]. This aligns with the broader clinical message from contemporary reviews of SVG failure: namely, that preventing early conduit injury is fundamental, as early thrombosis and inflammatory remodelling create the conditions for later intimal hyperplasia and accelerated atherosclerosis [2] (Table 2). Therefore, preservation should be regarded as a biologically active perioperative intervention that can influence subsequent graft susceptibility to thrombosis and remodelling, rather than as passive storage. Evidence that iatrogenic mechanical injury can generate specific and under-recognised failure modes further highlights the importance of meticulous surgical technique. Ren et al. described late SVG stenosis patterns associated with clipping metal side branches, which often occurs at corners or at multiple sites, creating resistance to the procedure during intervention. IVUS has clarified the mechanism and morphological pattern of these lesions [7]. Although these observations relate to a specific cause of graft failure, they reinforce the broader principle that standardising conduit handling and harvesting is essential, as advanced device-based or molecular therapies cannot fully compensate for avoidable surgical injury. While this mechanistic and preclinical framework helps to contextualise the clinical relevance of local graft protection strategies, it does not establish it. In particular, external support technologies have been developed to reduce adverse wall stress, improve luminal uniformity, and limit the haemodynamic triggers of pathological remodelling. Transcriptomic profiling of a biodegradable wrapping model revealed that external wrapping is associated with reduced inflammatory signalling and downregulation of pathways such as HIF-1, MAPK, NF-κB, TNF and IL-17. Meanwhile, the expression of genes associated with endothelial integrity and mechanotransduction, including PECAM1, NOS3 and KDR, remained relatively intact [9]. These findings are conceptually consistent with human spatial transcriptomic data obtained under arterial haemodynamics. This supports the idea that stabilising the graft mechanically and normalising wall shear stress may indirectly reduce early inflammatory programming [26]. A related biomaterial strategy builds on this concept by combining external support with local pharmacological modulation. Zhang et al. developed an electrospun external sheath that releases fasudil, everolimus and simvastatin slowly and sequentially over a period of weeks to months. This matches the phase-dependent evolution of SVG pathology. In an in vivo rabbit model, the drug-eluting sheath reduced intimal hyperplasia and improved intima/media ratios compared with both untreated controls and non-drug sheaths. However, mechanical restraint alone appeared insufficient to fully prevent disease progression [10]. Taken together, these studies provide a coherent rationale for translational research into peri-graft platforms that can act mechanically and pharmacologically, exploiting the conduit’s unique surgical accessibility. From a clinical perspective, however, interpreting local graft protection strategies requires caution. Many of these approaches were initially developed to improve graft biology and morphology by reducing intimal hyperplasia, limiting luminal irregularity and maintaining patency. However, early studies were often underpowered or not designed to detect differences in hard clinical endpoints. Consequently, the most informative evidence currently originates from larger observational comparisons and multicenter studies that report clinical composites in addition to surrogate angiographic or imaging endpoints [17]. The clinical outcome that appears most sensitive for external SVG support is repeat revascularisation, since the intervention acts locally on graft degeneration and is therefore more likely to affect graft-related events than mortality or stroke. This is demonstrated by the RESTART study, which is a propensity-matched international real-world comparison of VEST-treated patients and historical controls from the ART trial. In RESTART, major adverse cardiac and cerebrovascular events (MACCE) at one year were defined as the primary endpoint and included death, myocardial infarction lasting more than three days, any revascularisation and stroke. Secondary endpoints included five-year MACCE and target vessel revascularisation. The latter is particularly relevant because it is the event most directly attributable to graft failure.

Ref. [18] reported a significant reduction in MACCE at one year in the VEST group compared with controls (4.13% vs. 6.53%; HR 0.60, 90% CI 0.38–0.94), with a benefit that persisted at five years (16.43% vs. 21.82%; HR 0.70). Consistent with the underlying mechanical hypothesis, stroke rates were essentially unchanged, whereas the MACE composite excluding stroke showed a stronger five-year signal (13.91% vs. 19.31%; HR 0.67). Most notably, yearly risk of target vessel revascularisation (TVR) rates per person-year were significantly lower in the VEST group between years two and five, suggesting that the apparent clinical benefit was primarily driven by fewer revascularisations in previously venous territories, which is the endpoint most plausibly modifiable by a local graft protection strategy [17]. Additional non-randomised, multicentre data reported MACCE rates of 4.9%, 14.6% and 17.6% at one, three and five years, respectively, alongside revascularisation rates of 2.2%, 7.6% and 7.6%. This supports the interpretation of a possible benefit signal, although causality has not been established. The available clinical evidence for other external support platforms remains more limited and is not always derived from CABG populations. For instance, the FRAME device has primarily been evaluated in peripheral bypass surgery using a venous conduit in patients with chronic limb-threatening ischaemia and suboptimal graft quality. In a series of 16 patients treated between 2017 and 2023, the FRAME device achieved technical success and intraoperative patency in all cases. There was 30-day limb salvage and survival of 100%, primary patency of 93.7%, and no graft or device infections. During a median follow-up period of 32 months, primary patency was 68.7%, assisted primary patency was 75%, and overall limb salvage was 87.5%. Again, there was no evidence of device infection or aneurysmal dilatation of the supported veins [30]. Although these findings cannot be directly extrapolated to CABG, they support the broader concept that external venous support can safely be integrated into surgical reconstruction and may improve conduit behaviour in certain situations. Overall, the available evidence suggests that local graft protection is unlikely to rely on a single, universally effective intervention. Instead, a layered strategy combining atraumatic harvesting, optimised preservation and normalisation of the mechanical environment of the graft, as well as targeted local biological modulation where feasible, may offer the greatest potential for translation. This strategy should be implemented during the perioperative period, when the pathological process of vein graft disease begins [31].

## 6. Conclusions

The studies included in this review converge on one central point: VGD is initiated early, driven by mechano-transducive inflammation and remodelling, and uniquely accessible to local intervention during CABG. Human spatial transcriptomics demonstrates the rapid activation of inflammatory and remodelling pathways under arterial haemodynamics. Meanwhile, ex vivo NF-κB inhibition and microRNA-based local delivery studies demonstrate that molecular responses can be targeted within this timeframe. Further evidence that perivascular biomaterials can stabilise haemodynamics and deliver sustained therapy comes from external biodegradable wraps and drug-eluting sheaths. Taken together, these data support a translational framework for investigating whether perioperative conduit handling, preservation, external mechanical support and local RNA/drug delivery could mitigate the processes contributing to SVG failure [2,5,10,11].

## Figures and Tables

**Table 1:** Intraoperative and perioperative graft protection strategies.

Strategy	Study Type/Model	Main Mechanism of Local Protection	Main Findings Relevant to Graft Protection	Key Limitation
Buffered/endothelial-friendly preservation solutions [9,11,12,13]	Review of experimental and clinical literature	Reduces storage-related endothelial dysfunction Limits oxidative stress Preserves nitric oxide availability during ex vivo conduit preservation	Normal saline was associated with impaired endothelial-dependent and endothelial-independent vasodilation Buffered saline was associated with lower 1-year graft failure in cited literature The review also notes ongoing evaluation of DuraGraft	Evidence is mainly review-level Not a standalone original DuraGraft trial
No-touch/soft-touch harvesting [4,14]	Editorial/meta-analytic summary and review	Preserves perivascular fat Maintains vasa vasorum integrity Reduces endothelial trauma and conduit overdistension	Improved graft patency/failure rates versus conventional harvesting in pooled randomized data No clear reduction in clinical events Superior long-term patency reported in trials using no-touch technique	Hard clinical outcome benefit remains less consistent than angiographic benefit Open no-touch harvesting may increase leg wound complications
External metallic support (VEST) [15,16,17]	Randomised trial summary + prospective clinical study	Provides mechanical stabilization of SVGs Reduces wall tension and disturbed flow Improves lumen geometry	VEST III showed no significant difference in overall patency at 2 years Reduced intimal hyperplasia and improved angiographic profile In a 6–12 month prospective study, externally stented SVGs showed 98% patency and 90% lumen uniformity versus 87.5% and 37% in unsupported SVGs	Geometric and remodeling benefits appear stronger than patency benefit Long-term superiority remains unproven
Biodegradable external wrapping [10]	Original preclinical study; ovine carotid–jugular interposition vein graft model	Provides temporary external support during early arterial adaptation Allows gradual load increase Normalizes wall shear stress	Promoted luminal uniformity and more physiological wall shear stress Reduced overdistension, wall thickening, intimal hyperplasia, and inflammation Preserved mechanotransduction RNA data showed downregulation of inflammatory pathways	Preclinical large-animal evidence only Scalability and translation to coronary CABG require validation
External scaffold/support in non-coronary vein grafting [18]	Clinical observational study in CLTI infrainguinal bypass	Reinforces ectatic or varicose venous grafts Improves conduit geometry Prevents dilation and compression	Technical success and intraoperative patency were achieved in all cases No graft infection or reinforced-vein dilatation was reported Mid-term patency and limb salvage were acceptable	Not a CABG study Relevance is supportive and mechanistic rather than directly coronary
RNA-based local therapy (miR-126-3p agomir) [6]	Original translational study: human HSV endothelial cells, ex vivo HSV culture, rat vein arterialization model	Enhances re-endothelialization Suppresses neointimal hyperplasia Uses local graft-directed RNA delivery	Reduced ex vivo neointimal thickness Improved endothelial coverage Increased in vivo re-endothelialization to approximately 85–86% Reduced inflammatory cell infiltration Attenuated neointimal formation after local graft-confined delivery	Strong mechanistic rationale but still preclinical Clinical feasibility depends on delivery platform development
Intraoperative ex vivo graft conditioning (PREVENT IV; edifoligide + pressure-mediated delivery) and conduit-preservation insights [12,19]	Phase III randomised clinical trial in CABG patients; ex vivo pretreatment of SVGs before implantation	Local intraoperative transfection of the vein graft wall Intended to inhibit early neointimal hyperplasia during the perioperative window Related analyses highlighted the role of storage solution during ex vivo handling	Edifoligide did not reduce SVG failure or long-term clinical endpoints versus placebo Subsequent analyses showed that SVGs stored in buffered saline had lower 1-year failure rates than grafts stored in saline or blood A trend toward better 5-year outcomes was observed Findings suggest conduit handling and preservation may be at least as important as targeted antiproliferative delivery	Negative result for the biological agent itself Preservation-solution signal derives from secondary/post hoc analyses rather than the primary randomized comparison

**Table 2:** Summary of clinical evidence.

Study	Year	Design	Population	Intervention	Endpoints	Main Finding
PREVENT IV [12,19]	2005	Phase III, multicenter, randomised, double-blind, placebo-controlled trial	3014 patients, age 18–80 years, undergoing first isolated CABG with at least two planned saphenous vein grafts, enrolled at 107 US sites	Ex vivo treatment of autologous SVGs with edifoligide vs. placebo before implantation	Primary: SVG failure at 12–18 months, defined as death or ≥75% stenosis in a treated vein graft at angiographic follow-up Secondary: major adverse cardiac events through at least 5 years and adverse events through 30 days	Ex vivo edifoligide treatment did not reduce SVG failure No improvement in long-term clinical outcomes compared with placebo The trial supports the importance of conduit handling and preservation rather than edifoligide-based graft conditioning
VEST III [11]	2022	Prospective, within-patient, controlled, randomised, multicenter international trial	184 patients undergoing isolated CABG in 14 European centers, using an internal thoracic artery graft and at least 2 additional SVGs	One SVG randomised to external VEST stent, with one non-stented SVG as within-patient control	Primary: SVG Fitzgibbon patency scale by angiography at 2 years Secondary: intimal hyperplasia by IVUS in a prespecified subgroup at 2 years	Overall patency was similar between groups Externally stented grafts showed better Fitzgibbon grades Significant reductions in intimal hyperplasia area and thickness were observed Findings support a biological/mechanical effect on SVG remodeling, although long-term patency superiority remained unproven
RESTART [16]	2025	International observational comparative study with propensity-matched/weighted analysis; VEST-treated real-world cohort compared with historical ART controls	CABG patients receiving VEST-supported SVGs, compared with historical controls from the ART trial	Use of VEST external support on SVGs during CABG	Primary: MACCE at 1 year: death, myocardial infarction lasting >3 days, any revascularization, or stroke Secondary: 5-year MACE and target-vessel revascularization	VEST was associated with lower 1-year MACCE Benefit persisted at 5 years Strongest signal was observed for graft-related outcomes, especially repeat revascularization/target-vessel revascularization Stroke rates were essentially unchanged Findings suggest a clinical effect mainly driven by local graft protection rather than global cardiovascular risk reduction
Multicentric cohort on VEST [17]	2025	Multicenter, non-randomised cohort study	397 patients undergoing CABG in 3 centers, with at least one SVG supported by an external stent; 469 of 654 SVGs were stented	VEST-enhanced CABG in real-world practice, including on-pump/off-pump cases and some concomitant procedures	Follow-up for MACCE and repeat revascularization Outcomes reported at 1, 3, and 5 years Graft-territory revascularization compared between stented and non-stented SVG territories	Freedom from MACCE was 95.1%, 85.4%, and 82.4% at 1, 3, and 5 years Revascularization in territories grafted with stented SVGs was lower than in non-stented SVG territories: 1.28% vs. 4.32%, *p* = 0.015 Findings suggest feasibility, safety, and possible reduction in target-vessel revascularization Randomized causal proof is still lacking
FRAME clinical series [18]	2024	Observational clinical case series	16 patients treated from 2017 to 2023 for chronic limb-threatening ischemia with venous bypass using suboptimal/ectatic autologous conduit	Use of FRAME external vascular support for venous grafts in infrainguinal/peripheral bypass surgery	Technical success Intraoperative patency 30-day limb salvage and survival Primary and assisted primary patency during follow-up Device/graft infection Aneurysmal dilation	Technical success and intraoperative patency were 100% At 30 days, limb salvage and survival were 100%, with primary patency of 93.7% Over a median 32-month follow-up, primary patency was 68.7%, assisted primary patency 75%, and limb salvage 87.5% No graft/device infections or dilation of reinforced veins were reported Evidence supports feasibility and safety in peripheral bypass, but not direct CABG efficacy

## Data Availability

No new data were created or analyzed in this study. Data sharing is not applicable to this article.
